# Physical Activity Volume and Intensity for Healthy Body Mass Index and Cardiorespiratory Fitness: Enhancing the Translation of Children's and Adolescents' Accelerometer Physical Activity Reference Values

**DOI:** 10.1111/sms.70118

**Published:** 2025-08-19

**Authors:** Lynne M. Boddy, Alex V. Rowlands, Borja del Pozo Cruz, Sarah L. Taylor, Robert J. Noonan, Liezel Hurter, Matteo Crotti, Lawrence Foweather, Lee E. F. Graves, Owen Jones, Mhairi MacDonald, Deborah A. McCann, Caitlin Miller, Michael B. Owen, James R. Rudd, Richard Tyler, Stuart J. Fairclough

**Affiliations:** ^1^ The Physical Activity Exchange, Research Institute for Sport and Exercise Sciences Liverpool John Moores University Liverpool UK; ^2^ Diabetes Research Centre, Leicester General Hospital University of Leicester Leicester UK; ^3^ National Institute for Health Research (NIHR) Leicester Biomedical Research Centre (BRC) University Hospitals of Leicester NHS Trust and the University of Leicester Leicester UK; ^4^ Alliance for Research in Exercise, Nutrition and Activity (ARENA), UniSA Allied Health and Human Performance University of South Australia Adelaide Australia; ^5^ Department of Sport Sciences, Faculty of Medicine, Health, and Sports Universidad Europea de Madrid Madrid Spain; ^6^ Division of Public Health, Sport and Wellbeing, School of Allied and Public Health University of Chester Chester UK; ^7^ Department of Human and Social Sciences University of Bergamo Bergamo Italy; ^8^ Sport, Physical Activity, Health, and Wellbeing Research Group, Department of Sport and Physical Activity Edge Hill University UK; ^9^ International Centre for Applied Research With Children, Young People, Pregnant Women and Families (iCARE) Edge Hill University UK; ^10^ Department of Social Work and Wellbeing, Faculty of Health, Social Care and Medicine Edge Hill University UK; ^11^ Department of Teacher Education and Outdoor Studies Norwegian School of Sport Sciences Oslo Norway

**Keywords:** accelerometer, average acceleration, fitness, intensity gradient, obesity, overweight, youth

## Abstract

This secondary data analysis aimed to demonstrate the utility of physical activity (PA) wrist accelerometer outcome reference values by identifying the PA volume (average acceleration) and intensity distribution (intensity gradient) centiles and values associated with body mass index (BMI) status (normal weight, overweight, and obese) and cardiorespiratory fitness (CRF, multistage shuttle runs test) status (low, moderate, and high) in children and adolescents. We assessed the dose–response associations between average acceleration and intensity gradient with BMI and CRF outcomes using restricted cubic spline linear mixed models. To aid translation of the findings, we calculated the increases in average acceleration needed to shift exemplar participants to “healthy” weight and CRF status. For boys and girls, there was a nonlinear inverse association between average acceleration and BMI. In both sexes, a positive dose–response was observed between average acceleration and intensity gradient with CRF. The values and centiles of average acceleration and intensity gradient that aligned with BMI and CRF statuses were identified. To move from an average acceleration associated with overweight to healthy weight, 10‐year‐old boys and girls would need to increase daily average acceleration by 23 m*g* (~30‐min running) and 16 m*g* (~18‐min running), respectively. These findings further demonstrate the importance of PA in relation to BMI and CRF and the utility of PA reference values for the translation of accelerometer outcomes into meaningful information. Additional studies demonstrating how PA reference values can be used to track behaviors and provide insights into health associations could inform practice further.

## 1 Introduction

It is widely accepted that cardiorespiratory fitness (CRF) and body mass index (BMI) are strongly associated with current and future health status [1, 2]. Despite the wealth of evidence highlighting the importance of CRF and healthy body size for children's health, CRF assessed using field‐based multistage shuttle run tests has declined over recent decades [3]; the prevalence of obesity remains stubbornly high in the UK [4] and is increasing globally [5]. Physical activity (PA) is important to children's CRF and BMI because it is the modifiable contributor to CRF [2] and plays an important role in energy balance and regulation of eating behaviors [6].

Accelerometers have been used for many years to estimate PA and examine relationships between PA and health outcomes. Most of the existing research has used device‐specific outcomes and processed acceleration signals using absolute intensity cut‐points to estimate time spent inactive and engaging in light, moderate, and vigorous PA (LPA, MPA, and VPA, respectively) [7, 8, 9]. This approach is reliant on applying generic or population‐specific cut‐points that can lead to substantial differences in reported PA between studies, hindering comparisons or meaningful meta‐analyses [10, 11]. Consequently, there have been calls to move beyond proprietary processing methods and cut‐points to promote transparency, researcher‐driven decision making, and to aid comparability between studies [10, 12]. In recent years, the availability of raw acceleration signals and open‐source processing techniques has led to the development of device‐agnostic accelerometer outcomes and methods that allow clear representation of PA volume and intensity profiles [12, 13]. As time spent in MPA or VPA accounts for only small proportions of the day [10] and is highly correlated with PA volume [14, 15], the traditional cut‐point analyses approach makes it difficult to tease out the relative importance of PA volume and intensity for health. Conversely, cut‐point‐free outcomes such as average acceleration and intensity gradient provide a complementary picture of PA across the full day; therefore, no information is lost. Despite the availability and increased application of cut‐point‐free outcomes that represent the volume and intensity of PA, few studies have explored these outcomes alongside CRF or BMI in children. Of the limited available evidence, PA volume and intensity outcomes have demonstrated independent relationships with both CRF and BMI in 8–12‐yearyear‐old children [14, 16].

Reference values for accelerometer outcomes are a useful means of determining group‐level PA relative to population‐specific reference groups [17, 18]. Recently published UK child average acceleration and intensity gradient reference values [19] provide valuable comparative data for researchers wishing to benchmark their child accelerometer outcomes against a reference population. However, these reference values do not anchor PA to health outcomes, which limits their application. Addressing this limitation would allow researchers, clinicians, and PA practitioners to assess where children's PA outcomes intersect with health outcomes such as BMI and CRF, whether PA outcomes align with “healthy” thresholds for BMI and/or CRF, and to quantify the proportion of children who fall into the healthy categories for BMI and CRF. This aligns with the use of reference values by general practitioners (GPs) and in public health for other health‐related metrics, such as BMI and fitness (e.g., for BMI [20]). Therefore, the aim of this study was to demonstrate the utility of recently published PA accelerometer outcome reference values by identifying the PA volume and intensity centiles/values associated with healthy BMI and CRF in children and adolescents.

## 2 Materials and Methods

This study is a secondary analysis of a pooled harmonized dataset used to create reference values for wrist‐worn accelerometer volume and intensity PA outcomes in England for children and adolescents [19]. The data acquisition and harmonization processes have been described in detail elsewhere [19]. Briefly, ten ethically approved wrist accelerometry studies (7 cross‐sectional, 3 intervention studies) involving typically developing school‐aged youth (ranging from 5 to 15 years old) led or supervised by the first or last authors were identified for inclusion in the harmonized dataset. To be included, studies required nonintervention assessments of wrist accelerometer‐derived PA. For the included intervention studies, only baseline data were used. In addition to raw acceleration data, studies provided stature, body mass, and demographic data including age and sex. Where published, details of these studies can be found elsewhere [21, 22, 23, 24, 25, 26]. Data were available from *N* = 1250 children and adolescents (*N* = 510 boys, 720 girls, the sample sizes of the included studies ranged from *N* = 29 to 311. mean *N* = 150 ± 84) who participated in 10 studies conducted in 71 schools between 2015 and 2022 in the Merseyside, Lancashire, and Greater Manchester counties of northwest England. Ethical approval was granted by Edge Hill University's Science Research Ethics Committee (#ETH2021‐0034).

### 2.1 Anthropometric Outcomes

In all contributing studies, stature and body mass were measured following standard procedures to the nearest 0.1 cm/kg using a portable stadiometer and digital scales, with participants wearing light clothing and shoes removed [27]. Overweight and obese were defined as ≥ 85th percentile and ≥ 95th percentile, respectively, for BMI computed from UK 1990 reference data [20]. International Obesity Task Force age‐ and sex‐specific body mass index (BMI) cut‐points were also applied to classify participants as normal weight, overweight, or obese [28]. Age at peak height velocity (APHV) was calculated from anthropometric data using sex‐specific equations [29].

### 2.2 Cardiorespiratory Fitness

Two studies (*N* = 160 girls, 147 boys; Age = 10.0 ± 0.4 years) included within the harmonized dataset also provided CRF data, estimated using the field‐based 20‐m shuttle run test (20mSRT). The test has been widely used with participants aged the same as those within this study [30]. To allow comparison with international CRF studies, the number of completed laps, end running speed, and estimated peak oxygen uptake (VO _2peak_; ml‧kg‧min^−1^) using the Leger et al. prediction equation [31] were included in this analysis. Using a normative quintile‐based framework, children were classified as having “high,” “moderate,” or “low” CRF levels based on the 60th and 40th centile cut‐offs for 20mSRT‐estimated VO_2peak_ from over 1 million children [3].

### 2.3 Physical Activity

In the contributing studies, ActiGraph GT9X (ActiGraph, Pensacola, FL; 8 studies) or GENEActiv Original (Activinsights, Cambs, UK; 2 studies) triaxial accelerometers were used. The devices have a dynamic range of ±8 *g* and were requested to be worn for up to 7 consecutive days on the nondominant wrist using either 24‐h (8 studies) or waking hours wear protocols (2 studies), with a sampling frequency set at 100 Hz (8 studies) or 30 Hz (2 studies). The devices were initialized and data downloaded using the latest releases of the respective ActiLife (versions 6.13.1 to 6.13.4) and GENEActiv (versions 2.2 to 3.1) available at the time of data collection. PA outcomes were generated from the raw accelerometer data files (ActiGraph: gt3x then conversion to .csv format; GENEActiv: bin format) and were processed in R using package GGIR version 2.6–0 [32].

### 2.4 Accelerometer Data Harmonization

Data from 24‐h and waking hours protocols were harmonized by defining the age and day‐specific waking windows of interest as described in detail elsewhere [19]. These age‐ and day‐specific averaged waking and sleep times were then used in separate GGIR shell R scripts (*qwindows* argument). Age group and week/weekend day accelerometer files were then reprocessed separately in GGIR part 2 to calculate the waking hours PA acceleration outcomes. Signal processing included autocalibration using local gravity as a reference [33], detection of implausible values, and detection of nonwear. Invalid data were imputed by the average at similar time points on other days of the week as default in GGIR [34]. Wear time criteria were at least three valid weekdays and one valid weekend day, with a valid wear day defined as ≥ 600 min·d^−1^ of accelerometer wear during waking hours. Participants' accelerometer data were excluded from analyses if the wear time criteria were not achieved and/or postcalibration error was > 10 m*g* (milli‐gravitational units).

### 2.5 Accelerometer Outcomes: PA Volume and Intensity

PA volume: Average acceleration (i.e., average magnitude of dynamic acceleration) was calculated during GGIR part 1 processing using ENMO (i.e., the Euclidean norm of the three accelerometer axes with 1 *g* subtracted and negative values truncated to zero [34]) averaged over 1‐s epochs. Epochs were expressed in m*g* and averaged over the waking day to represent PA volume.

PA intensity distribution: The intensity gradient was calculated in GGIR part 2. This metric describes the distribution of the intensity of an individual's PA during the measurement period and reflects the negative curvilinear relationship between intensity and time accumulated at any given intensity [15]. A higher intensity gradient (i.e., less negative value) reflects proportionately more time being spread across the intensity profile, whereas a lower or more negative gradient reflects proportionately less time spent in mid‐range and higher intensities. Intensity gradient was expressed over the waking day.

### 2.6 Analysis

Processed accelerometer data for each age group were first combined and then average weekly values computed, weighted for weekdays and weekend days (5:2 ratio), for average acceleration and intensity gradient. Using each participant's unique ID code, these data were harmonized with the corresponding anthropometric, demographic, BMI, and CRF data, after which sex‐ and age‐group descriptive statistics were calculated. We assessed the dose–response associations between PA volume (average acceleration) and intensity (intensity gradient) with BMI and CRF outcomes using restricted cubic spline linear mixed models to allow for potential nonlinearity. For this analysis, we trimmed observations less than 1% and greater than 99% of the distribution. We prespecified knots placed at the 10th, 50th, and 90th percentiles of the exposure distribution. We assumed linearity for values below the 10th percentile and for values above the 90th percentile. Departure from linearity was assessed by a Wald test examining the null hypothesis that the coefficient of the second spline was equal to zero. Analyses were stratified by sex and adjusted for age, APHV, season of data collection, and school type (primary or secondary; BMI analysis only). Models for BMI and CRF‐related outcomes were mutually adjusted for each other. Results were plotted to identify the range of PA volume and intensity values and centiles related to weight status and CRF status classifications. All analyses were conducted in R (version 4.4.1) using packages *rms* for testing associations and *ggplot* for visualization.

To aid translation of the findings, we calculated the increase in average acceleration needed to shift participants from the centile/value associated with overweight and low fitness status to “healthy” weight and CRF status zones by adding time spent in slow (3 km/h) and fast (5 km/h) walking and running (8 km/h). These everyday activities were chosen because corresponding average acceleration reference values have been previously reported for them [35] (see File S1 for calculations).

## 3 Results

The descriptive characteristics and outcomes for the participants are detailed in Table 1. Mean age was 10.3y ±2.4, with girls 0.9y older than boys. The highest proportion of participants was in the 8–10y age group, with the lowest proportion aged 5–7 years. Mean BMI was 18.9 kg·m^2^ ±3.7, and around 73% of boys and girls were classified as healthy weight. CRF data were available from 307 participants aged 10.0y ±0.4. Of these, almost half had high CRF, and around a third had low CRF. Relatively more girls than boys were in the high or moderate CRF classifications. Accelerometers were worn for an average of 14.1 h·day^−1^ ±0.9 over 6.1 days ±0.8. Average acceleration and intensity gradient were highest among boys, indicating that their PA volume and intensity distribution were greater than girls by 21.1% and 7.2%, respectively.

**TABLE 1:** Participants' descriptive characteristics and outcomes (Mean (SD), unless stated otherwise).

	All	Boys	Girls
*n*	1250	510	740
Age (y)	10.3 (2.4)	9.8 (2.0)	10.7 (2.5)
Age group (%)			
5–7 y	14.2	16.1	12.8
8–10 y	36.3	39.4	34.2
11–12 y	25.4	32.0	20.9
13–15 y	24.1	12.5	32.0
Height (cm)	141.9 (15.2)	139.3 (14.0)	143.7 (15.6)
Weight (kg)	39.2 (13.9)	36.3 (12.3)	41.1 (14.9)
APHV (y)	−2.4 (2.8)	−3.5 (2.1)	−1.7 (2.9)
BMI (kg·m^2^)	18.9 (3.7)	18.2 (3.4)	19.3 (3.9)
BMI‐z	0.51 (1.2)	0.50 (1.2)	0.52 (1.2)
Weight status (%)			
Healthy weight	73.4	73.7	73.2
Overweight	18.7	19.1	18.5
Obese	7.9	7.2	8.4
Accelerometer outcomes			
Valid wear days	6.1 (0.8)	6.2 (0.8)	6.1 (0.7)
Valid wear time (h·day^−1^)*	14.1 (0.9)	14.0 (0.8)	14.2 (0.9)
Average acceleration (m*g*)*	63.2 (21.8)	72.2 (23.8)	57.0 (17.9)
Intensity gradient*	−2.16 (0.18)	−2.07 (0.1)	−2.22 (0.17)
CRF sample *n*	307	147	160
Age (y)	10.0 (0.4)	10.0 (0.4)	10.0 (0.4)
20mSRT laps	30.7 (15.8)	33.3 (16.8)	27.8 (13.6)
20mSRT speed (km·h^−1^)	10.1 (0.9)	10.2 (0.9)	10.0 (0.8)
VO_2peak_ (ml·kg·min^−1^)	46.8 (4.2)	47.4 (4.6)	46.1 (3.7)
CRF status (%)			
High	46.3	45.6	46.9
Moderate	18.6	15.6	21.3
Low	35.2	38.8	31.9

*Note:* 20mSRT = 20 m shuttle run test; VO_2peak_ = estimated peak maximal oxygen uptake.

Abbreviations: APHV, age at peak height velocity; BMI, body mass index; CRF, cardiorespiratory fitness; m*g*, milligravitational units.

* Average acceleration and intensity gradient are expressed over the waking day.

### 3.1 BMI

**FIGURE 1:**
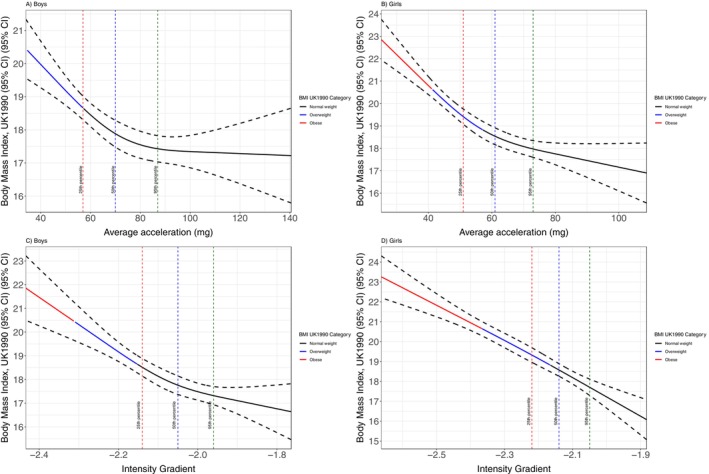
(A–D) Relationships between BMI and weight status with average acceleration and intensity gradient in boys and girls. The red, blue, and green dashed lines represent the previously published reference values for the 25th, 50th, and 95th centiles, respectively, for average acceleration (A, B) and intensity gradient (C, D) [19]. The red, blue, and black sections of the curve represent obese, overweight, and normal weight BMI status, respectively.

For both boys and girls, there was a nonlinear inverse association between average acceleration and BMI (Figure 1A,B). The BMI curve declined steeply with increasing average acceleration up to about the 50% centile of average acceleration (boys: 71 m*g*, BMI = 17.87; girls: 61 m*g*, BMI = 18.53), after which the drop was shallower. This plateau was more evident for boys. For boys' intensity gradient, the association with BMI was reflected by the curve becoming less steep from around the 25th centile (−2.14, BMI = 18.51). The girls' intensity gradient‐BMI curve was linear, with a slight deviation from this observed at the 95% centile (−1.94, BMI = 16.64).

### 3.2 BMI Classifications

The previously published PA reference values for average acceleration and intensity gradient [19] were presented for the 3rd, 10th, 25th, 50th, 75th, 90th, 95th, and 97th percentiles. For this study, the thresholds for healthy weight and fitness status are approximated with the above percentiles to ease translation and interpretation. When UK 1990 BMI reference data weight status cut‐points [20] were applied to the boys' PA outcomes, the healthy weight/overweight threshold was observed at approximately the 25th centile for average acceleration (57 m*g*) and intensity gradient (–2.15; Figure 1 and Table 2). Boys' BMI cut‐offs for obesity corresponded to average acceleration and intensity gradient values of 34 m*g* and −2.31 (both approximate to the 3rd centile). The girls' BMI threshold for healthy weight aligned approximately to the 50th centile for average acceleration (57 m*g*) and intensity gradient (−2.16), whereby the 5th and 3rd centiles for average acceleration (41 m*g*) and intensity gradient (−2.26), respectively, reflected the BMI cut‐off for obesity (Figure 1C,D).

**TABLE 2:** Weight status and CRF status thresholds corresponding to average acceleration and intensity gradient centiles.

	Boys		Girls	
	Average acceleration (approximate centile/m*g*)	Intensity gradient (approximate centile/value)	Average acceleration (approximate centile/m*g*)	Intensity gradient (approximate centile/value)
Weight status				
Healthy weight	> 25th/> 57	> 25th/> −2.15	> 50th/> 57	> 50th /> −2.16
Overweight	25th/57	25th/2.15	50th/57	50th/2.16
Obese	3rd/34	3rd/2.31	5th/41	3rd/2.26
CRF status				
High	95th/123	97th/1.80	95th/95	95th/1.94
Moderate	50th/76	50th/2.14	50th/60	50th/2.15
Low	25th/56	25th/2.16	3rd/33	5th/2.31

*Note:* Average acceleration and intensity gradient values are approximated to the closest published centile reference value. Abbreviations: CRF = cardiorespiratory fitness; m*g* = milligravitational units.

### 3.3 CRF

**FIGURE 2:**
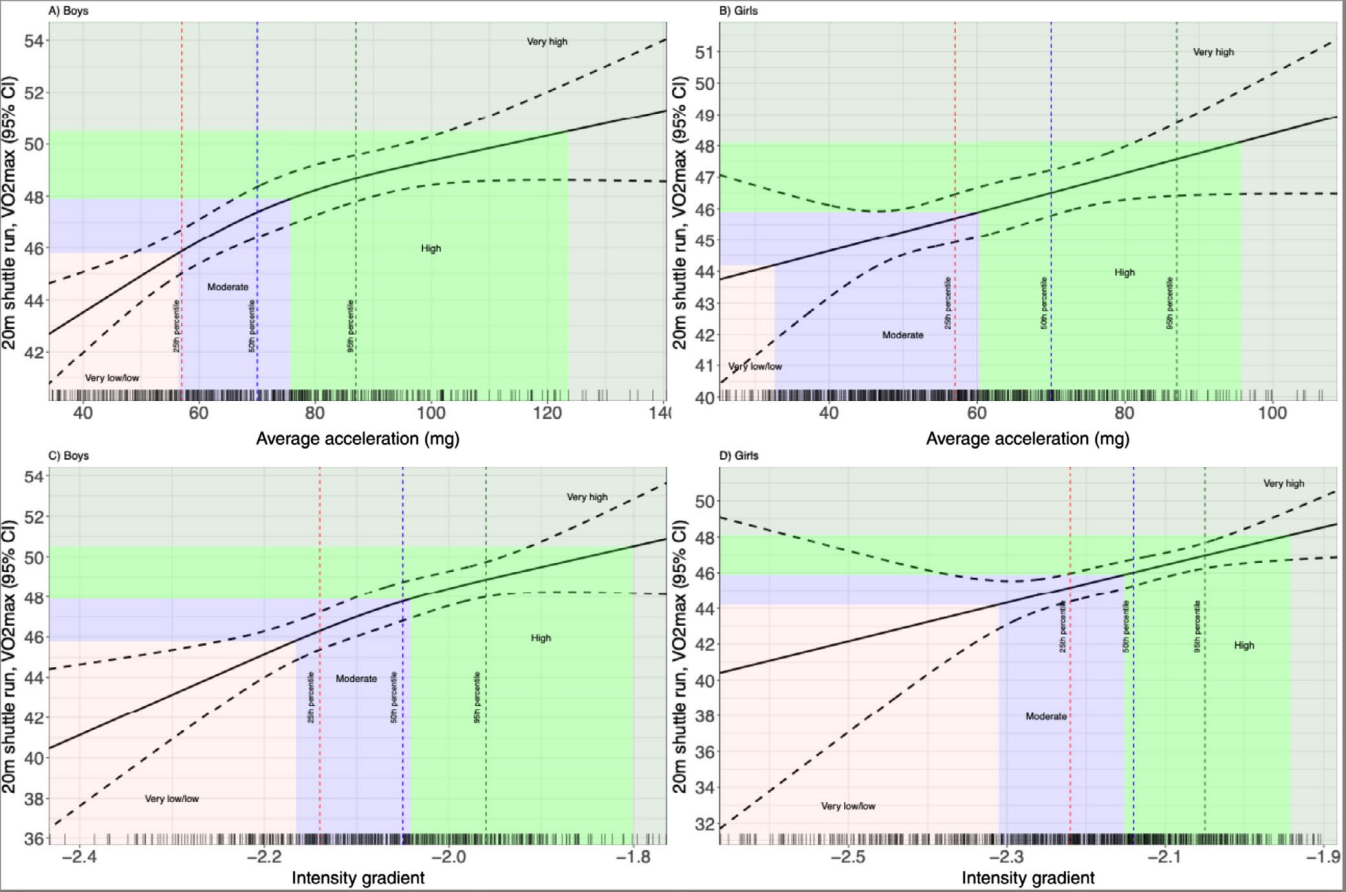
(A–D) Relationships between VO_2peak_ and CRF status with average acceleration and intensity gradient. The red, blue, and green dashed lines represent the previously published reference values for the 25th, 50th, and 95th centiles, respectively, for average acceleration (A, B) and intensity gradient (C,D) [19].

A positive dose–response was observed for boys and girls for the associations between both average acceleration and intensity gradient with VO_2peak_ (Figure 2), 20mSRT laps, and 20mSRT speed. For boys, the VO_2peak_ curve became curvilinear at the 50th centile of average acceleration (76 m*g*, VO_2peak_ = 47.40 mL·kg·min^−1^) and the 25th centile of intensity gradient (−2.16, VO_2peak_ = 46.30 mL·kg·min^−1^) (Figures 2A–D). A similar pattern was evident when 20mSRT laps and speed were plotted against the 2PA outcomes (File S2). Among girls, the curves representing the relationships between average acceleration and intensity gradient with CRF outcomes were typically characterized by being flatter and more linear than the boys'. The 50th centile of average acceleration and intensity gradient corresponded to values of 60 m*g* (VO_2peak_ = 45.94 mL·kg·min^−1^) and −2.15 (VO_2peak_ = 46.00 mL·kg·min^−1^), respectively.

### 3.4 CRF Classifications

Table 2 and (Figure 2C,D) show that average acceleration values below the ~25th (56 m*g*) and ~3rd (33 m*g*) centiles were associated with low CRF (VO_2peak_) among boys and girls, respectively. The ~50th (boys = 76 m*g*, girls = 60 m*g*) and ~95th (boys = 123 m*g*, girls = 95 m*g*) centiles for average acceleration aligned to moderate and high CRF in boys and girls. Low, moderate, and high CRF corresponded approximately to the 25th, 50th, and 97th centiles of boys' intensity gradient, respectively. The intensity gradient thresholds were lower among girls, with low, moderate, and high CRF falling at the respective ~5th, ~50th, and ~95th centiles of the distribution.

### 3.5 Translation

**FIGURE 3:**
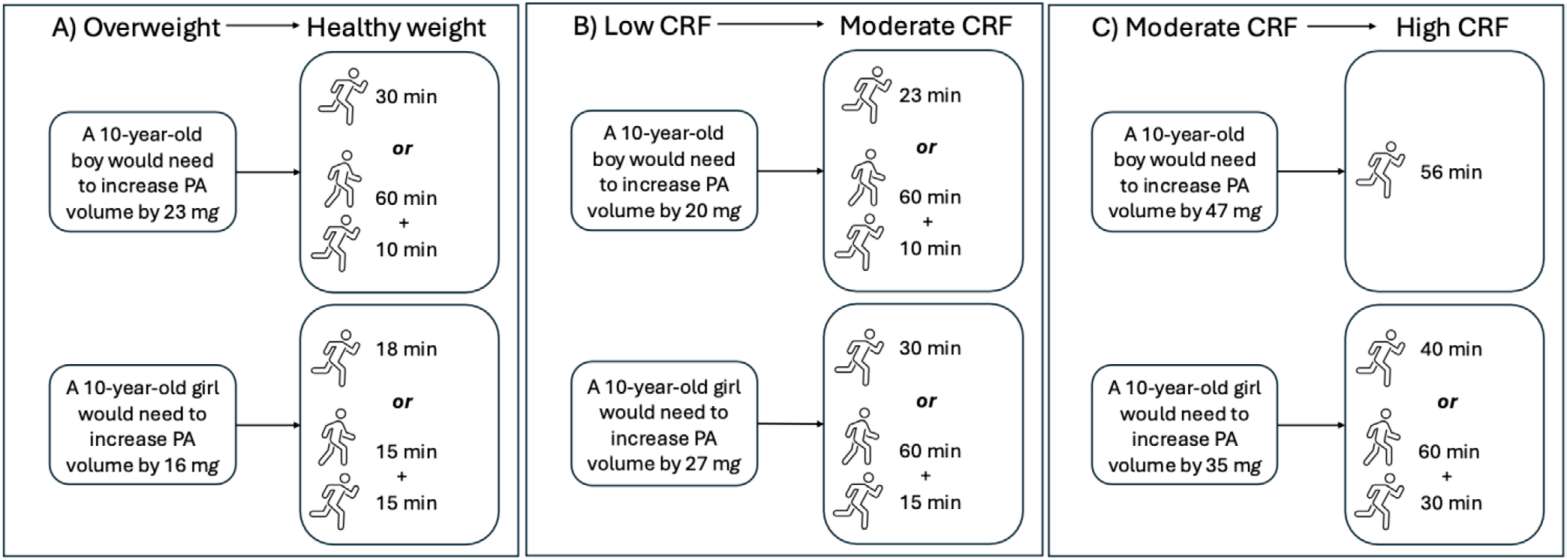
(A–C), the volume of PA accrued over a day (average acceleration, m*g*) required to move from the acceleration associated with overweight to healthy weight (a), low‐to‐moderate CRF (b), and moderate‐to‐high CRF (c) categories in 10‐year‐old boys and girls, using the examples of running at 8^−1^ km·h and fast walking at 5 km·h^−1^. Icons from Microsoft Word 365 Stock Images.

To enhance the translation of these findings in the context of improving weight status and CRF status through everyday activities, we have presented some simple scenarios here using previously published values for average acceleration [35] and the centiles/values identified in Table 2 (Figure 3A). For example, for an overweight 10‐year‐old boy to achieve an average acceleration associated with healthy weight status, he would need to increase his daily PA volume (average acceleration) by 23 m*g* (i.e., from 34 m*g* which corresponds to the threshold of upper threshold for obesity/lower threshold for overweight to 57 m*g*, which is the threshold for healthy weight, see Table 2 for centiles/values). This could be achieved through accumulating 30‐min running at 8 km·h^−1^, or approximately 60 min of fast walking (5 km·h^−1^) plus 10 min of running across the day. For an overweight 10‐year‐old girl, a PA volume increase of 16 m*g* (i.e., from 41 m*g*, the upper threshold for obesity/lower threshold for overweight to 57 m*g* which is the lower threshold for healthy weight, see Table 2) would be needed to cross into the healthy weight zone. Combinations of accumulating 15‐min running plus 15‐min fast walking, or 10‐min running, 30‐min fast walking plus 30‐min slow walking at 3 km·h^−1^, or approximately 18‐min running could be integrated across the day to achieve this. Similarly, to move from the average acceleration associated with the low to the moderate CRF category, 10‐year‐old boys and girls would need to increase daily PA volumes by 20 m*g* and 27 m*g*, respectively (Figure 3B). This could be the equivalent of accumulating 23 min (boys) and 30 min (girls) of 8 km·h^−1^ running or combining 60‐min 5 km·h^−1^ fast walking with shorter durations of running (boys: 10 min, girls: 15 min). The required daily PA volume to transition from values associated with moderate‐to‐high CRF (Figure 3C) would be 47 m*g* and 35 m*g* for boys and girls, respectively. This increase could be reflected by accumulating an additional 56 min of running for boys, and for girls either 40 min of running or 30 min of running plus 60 min of fast walking.

## 4 Discussion

This study aimed to demonstrate the utility of recently published PA accelerometer outcome reference values [19] by identifying the PA volume and intensity centiles and values associated with healthy BMI and CRF in children and adolescents. We observed the strongest dose–responses between BMI and PA volume and intensity in the least active children. The distributions of PA outcomes across weight status categories were less consistent between the sexes as healthy weight corresponded to the ~25th centile of average acceleration and intensity in boys and the ~50th centile in girls. CRF in boys was more strongly associated with lower levels of PA volume and intensity, whereas the dose–responses among girls followed a more linear trajectory. Boys and girls with greater than the median average acceleration and intensity gradient had high or very high CRF status; however, a greater proportion of boys than girls were classified as having low or very low CRF.

### 4.1 BMI and Weight Status

The nonlinear inverse associations between BMI and average acceleration demonstrated steep declines with increasing acceleration up to the ~50th centile for boys and girls, thus showing the strongest dose–response in the least active children. This suggests that small increments in the volume of daily PA were beneficially associated with BMI. This is consistent with evidence in adults that a 1 m*g* change in average acceleration is a clinically important difference for health benefits in inactive adults [36]. The associations with intensity gradient differed between boys and girls, with the boys' curve demonstrating strongest dose–responses in the least active children, becoming less steep around the 25th centile. Conversely, the more linear curve observed for girls suggests a largely consistent pattern across the intensity distribution. Previous research that has examined the association between BMI and time spent in MPA, VPA, and MVPA classified using the absolute cut‐point method and the more contemporary intensity spectrum approach (e.g., [37, 38, 39]) has outlined the inverse associations between PA intensity and BMI. For example, compositional isotemporal substitution analyses of children's wrist accelerometer data showed that time spent in MVPA was more predictive of favorable changes in BMI z‐scores than LPA or other movement behaviors [14, 38], while our previous study showed that PA volume reflected by time spent with average accelerations ≥ 700 m*g* was inversely associated with BMI z‐score [39]. Taken together, these findings suggest that higher intensity PA may be particularly important when present in small amounts, that is, in those with a low PA volume or intensity gradient. Further, these cross‐sectional findings are consistent with longitudinal data illustrating the importance of VPA for future healthy weight status [37]. Moreover, evidence from a recent systematic review highlights the beneficial effect of high‐intensity exercise interventions on body composition outcomes and that greater increases in cardiorespiratory fitness are associated with high‐intensity rather than moderate‐intensity exercise interventions in children and adolescents [40].

When examining weight status categories, the boys' thresholds for healthy weight were observed at the ~25th centiles of the reference values for average acceleration (57 m*g*) and intensity gradient (−2.15). Boys' thresholds for obesity fell at the ~3rd centile, equating to average acceleration and intensity gradient values of 34 m*g* and −2.31, respectively. The girls’ healthy weight threshold aligned to the ~50th centiles for average acceleration and intensity gradient, but the corresponding values of 57 m*g* average acceleration and −2.16 intensity gradient were similar to the boys. It is widely accepted that girls are less active than boys at all ages [41]. Moreover, given the vital role of PA in energy balance, it is therefore unsurprising that the girls' PA volume and intensity distribution corresponding to healthy weight fell at a higher percentile value than boys. In contrast, the girls' obesity thresholds aligned to the ~5th centile for average acceleration (41 m*g*) and ~3rd centile for intensity gradient (−2.36), which approximated to the equivalent values in boys.

### 4.2 CRF and CRF Status

For CRF, similar dose–response associations to the BMI analysis were observed for boys' and girls' PA volume and intensity outcomes, with girls' data characterized by a more linear and flatter curve. The girls' findings indicate the beneficial influence of time spent in higher intensities of PA regardless of habitual PA levels, while for boys, engaging in higher intensities of PA may be relatively more beneficial for CRF among those in the lowest quartile of PA volume and intensity distribution.

Average acceleration values below the ~25th and ~3rd centiles were associated with low CRF among boys and girls, respectively, while moderate and high CRF fell at the~50th and ~95th centiles of average acceleration for both boys and girls. Low, moderate, and high CRF corresponded to the ~25th, ~50th, and ~97th centiles of boys' intensity gradient, respectively, compared to the ~5th, ~50th, and ~95th centiles of the girls' distribution. As girls were less active than boys, lower average acceleration and intensity gradient values were observed for girls at equivalent centile values as well as for the threshold centiles for low and high CRF. If causal, this would suggest that boys require greater PA volume and intensity PA to achieve low, moderate, or high CRF in comparison to girls. Boys routinely have higher CRF across childhood and adolescence [3] and this sex difference is therefore reflected in the greater PA volume and intensity required to achieve a relative classification of fitness in comparison to girls. Of note, the centiles and corresponding average acceleration and intensity gradient values for moderate CRF were very similar to those for healthy weight in girls, suggesting that targeting the 50th centile for both average acceleration and intensity gradient would correspond with moderate fitness and healthy weight in girls (Table 2).

To aid the translation of these findings, we have presented illustrative data for typical physical activities using the example of 10‐year‐old boys and girls. For a 10‐year‐old boy to move from an average acceleration that corresponds with overweight to an average acceleration that corresponds with healthy weight, he would need to increase his average acceleration by 23 m*g*, which is the equivalent of accumulating an additional 30 min of running across the day, or a combination of 60 min of brisk walking plus 10 min of running across the day. This is slightly more than the PA recommendations for children of 60 min MVPA per day [42], consistent with the greater amount of PA needed to lose weight in adults than to maintain a healthy weight [43]. This provides practical information that could be used by practitioners to inform PA programming or group‐level PA prescription. To extend these illustrative examples, practitioners could also estimate time required using other activities of similar intensities based on published energy expenditure values. For example, playing tag instead of walking at 5 km/h or swapping running for swimming front crawl [44], thus enhancing the translation of the PA reference values for children, practitioners, and parents/guardians.

This is the first study to apply average acceleration and intensity gradient reference values to health outcomes in children and adolescents. Strengths of the study include using age‐group‐specific 24‐h data to establish waking and sleep times, which allowed the PA outcome to reflect actual waking hours durations. There was a high level of compliance with the accelerometer wear protocol, with data available from 83% of participants who had some recorded accelerometer outcome data. This exceeded the compliance level reported in other large‐scale child accelerometer data pooling studies [18]. Moreover, we used empirically derived BMI [20] and CRF thresholds [3] that were specific to the study population and assessment methods used, thus enhancing the ecological validity of our results. Study limitations include the use of cross‐sectional data generated from ten studies, with an unequal age distribution of participants who were not representative of English youth in general, and accelerometer data were collected across different periods, which may have resulted in seasonal variation in movement behaviors between the studies. Further, the CRF data were only available in two of the studies, further reducing the generalizability for those outcomes. No *a priori* power calculation was completed for this study, as is often the case with secondary data analyses from pooled studies. We presented CRF data as estimated VO_2peak_ using widely validated and used equations to aid comparability with other studies. There are known biases with estimated VO_2peak_; therefore, we also presented outcomes in Supporting Information (File S2) for end running speed and number of completed laps. While average acceleration and intensity gradient values are compared to BMI and CRF outcomes, including classifications for healthy weight and fitness zones, these data are cross‐sectional, and causality cannot be conferred. Furthermore, we chose BMI as a health outcome, as it is arguably the most widely used body size outcome within the existing research and, as such, provides utility for the discipline. Nevertheless, BMI is a measure of body size rather than composition, and the proportions of lean and fat mass vary throughout childhood and adolescence; despite using thresholds that were developed for children and adolescents, there may have been some misclassification of some of the participants. We used the examples of a 10‐year‐old boy and girl to help translate the findings into meaningful information for research users because this age represents the mean of the overall study sample. However, future studies applying the PA outcome reference values to BMI and CRF data may choose the present examples across the additional age ranges.

## 5 Conclusion

This study demonstrates the utility of PA reference curves in relation to markers of health in children and adolescents. The dose‐response curves demonstrate the importance of PA in relation to BMI and CRF. Translation of accelerometer outcomes into meaningful information can provide practitioners with practical examples to inform programming and practice. Future studies demonstrating how PA reference values can be used to track behaviors and/or provide insights into associations with health are needed to further advance the field and demonstrate how cut‐point free measures of accelerometer‐assessed PA can be meaningful.

## 6 Perspective

The prevalence of obesity and overweight remains high in children and adolescents, while levels of cardiorespiratory fitness (CRF) have declined. Physical activity (PA) is favorably associated with body mass index (BMI) and CRF. Advances in the processing of accelerometer‐assessed PA have led to outcomes that represent the overall volume (average acceleration) and intensity distribution (intensity gradient) of PA. Reference values for these metrics were recently published but are difficult to translate into meaningful health outcomes for practitioners. This study demonstrates the utility of the PA accelerometer outcome reference values by identifying the average acceleration and intensity gradient centiles and values associated with BMI and CRF status in children and adolescents. The study also describes how much additional PA a 10‐yr‐old boy and girl would need to accrue over the course of a day to move from values associated with overweight and low CRF to values associated with healthy BMI and moderate or high CRF, thus providing practical information that could be used by practitioners to inform PA programming. This study highlights the importance of PA in relation to children s BMI and CRF and the utility of PA reference values for use in practice.

## Author Contributions

L.M.B., S.J.F., A.V.R., and B.d.P.C. designed the study. S.J.F., L.M.B., M.C., L.F., L.H., O.J., M.M., D.A.M., C.M., R.J.N., M.B.O., S.L.T., and R.T. contributed to data collection in the original studies. B.d.P.C., S.J.F., and A.V.R. analyzed the data. L.M.B., S.J.F., A.V.R., and B.d.P.C. drafted the manuscript. All authors contributed to writing, editing, reviewing, and approved the final manuscript. Funding for the original studies, where applicable, was acquired by S.J.F., M.M., and R.T.

## Ethics Statement

Ethical approval for this pooled individual participant data study was granted by Edge Hill University's Science Research Ethics Committee (#ETH2021‐0034).

## Consent

Each participating study received ethical approval, and all participants had parental/carer written informed consent.

## Conflicts of Interest

The authors declare no conflicts of interest.

## Supporting information


**File S1:** sms70118‐sup‐0001‐Supplementaryfile1.xlsx.

**File S2:** sms70118‐sup‐0002‐Supplementaryfile2.docx.

## Data Availability

The data that support the findings of this study are openly available in the Open Science Framework at https://osf.io/5dy9k/files/osfstorage.
