# Supplementary material for: Physical Activity Volume and Intensity for Healthy Body Mass Index and Cardiorespiratory Fitness: Enhancing the Translation of Children's and Adolescents' Accelerometer Physical Activity Reference Values
Source: Scand J Med Sci Sports. 2025 Aug 19;35(8):e70118. doi: 10.1111/sms.70118 (PMC12363382; doi:10.1111/sms.70118)
Supplement: Supplementary file 2 — File S2: sms70118‐sup‐0002‐Supplementaryfile2.docx. [file SMS-35-e70118-s001.docx]

***Supplementary File 2***


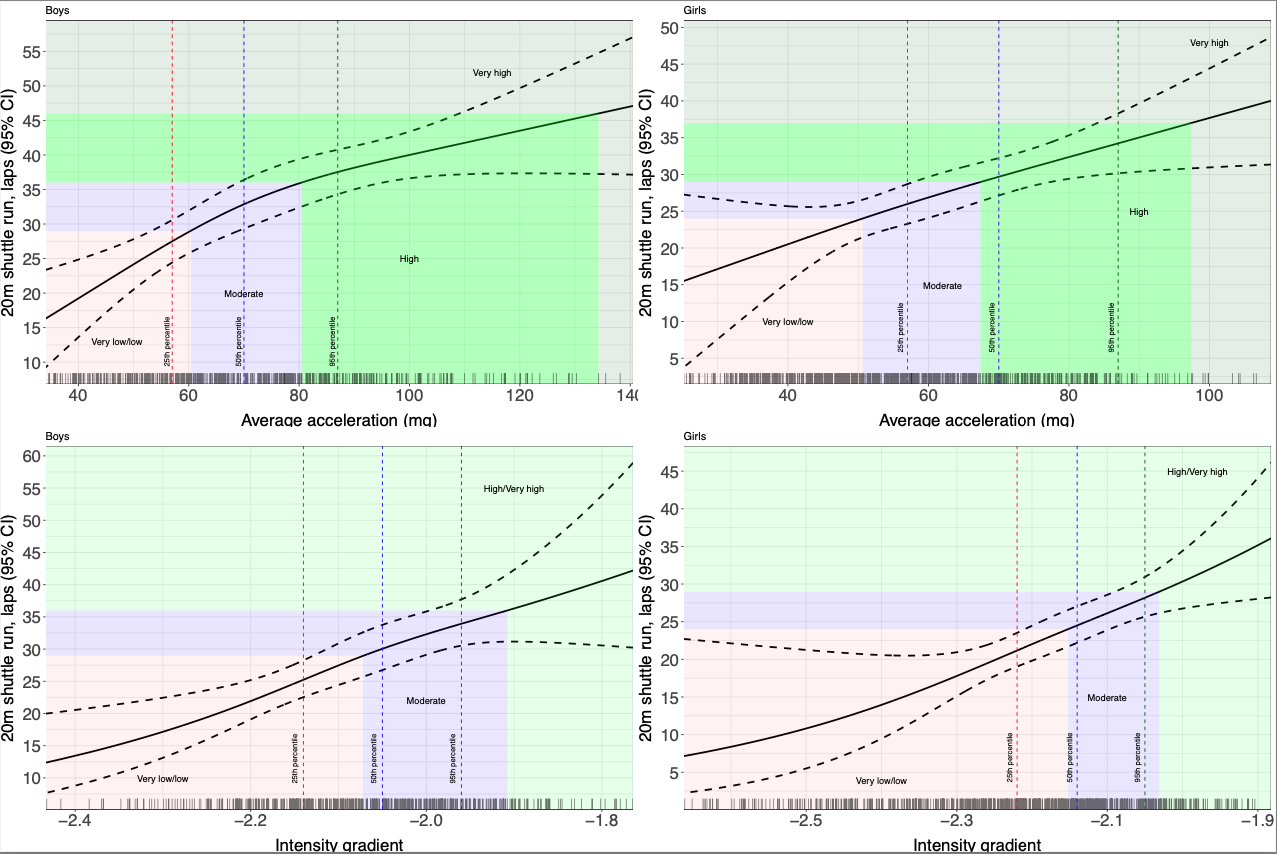


Relationships between number of completed laps and CRF status with average acceleration and intensity gradient. The red, blue and green dashed lines represent the previously published reference values for the 25^th^, 50^th^ and 95^th^ centiles respectively for average acceleration (A, B) and intensity gradient (C,D)^16^.


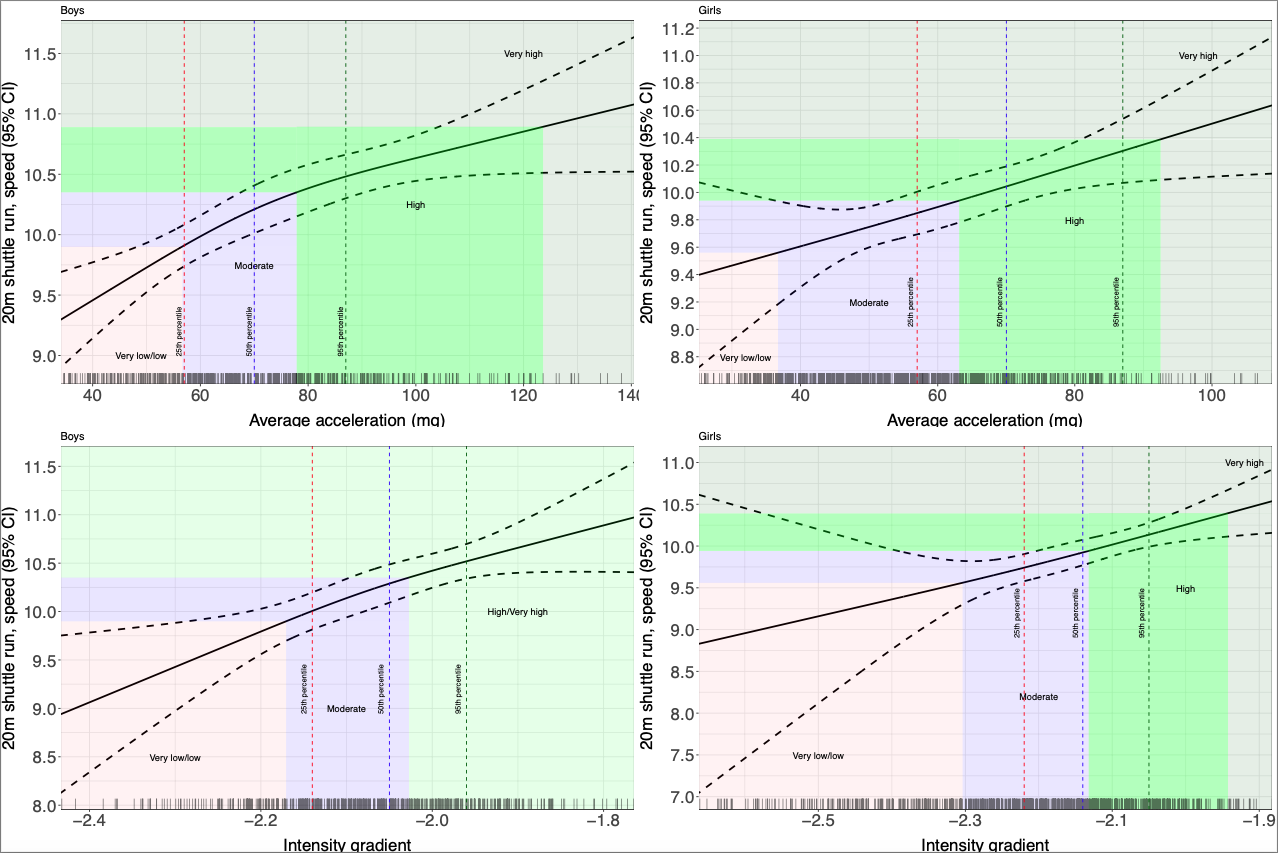


Figure 2a-d. Relationships between end running speed and CRF status with average acceleration and intensity gradient. The red, blue and green dashed lines represent the previously published reference values for the 25^th^, 50^th^ and 95^th^ centiles respectively for average acceleration (A, B) and intensity gradient (C,D)^16^.
